# Oxidative Stress and Inflammatory Status in COVID-19 Outpatients: A Health Center-Based Analytical Cross-Sectional Study

**DOI:** 10.3390/antiox11040606

**Published:** 2022-03-22

**Authors:** Sahar Golabi, Sheyda Ghasemi, Maryam Adelipour, Reza Bagheri, Katsuhiko Suzuki, Alexei Wong, Maryam Seyedtabib, Mahshid Naghashpour

**Affiliations:** 1 Department of Medical Physiology, School of Medicine, Abadan University of Medical Sciences, Abadan 6313833177, Iran; s.golabi@abadanums.ac.ir; 2 School of Medicine, Abadan University of Medical Sciences, Abadan 6313833177, Iran; shaydagh1998@gmail.com; 3 Department of Biochemistry, School of Medical Sciences, Ahvaz Jundishapur University of Medical Sciences, Ahvaz 6135715794, Iran; adelipour-m@ajums.ac.ir; 4 Department of Exercise Physiology, University of Isfahan, Isfahan 8174673441, Iran; will.fivb@yahoo.com; 5 Faculty of Sport Sciences, Waseda University, 2-579-15 Mikajima, Tokorozawa 359-1192, Japan; katsu.suzu@waseda.jp; 6 Department of Health and Human Performance, Marymount University, Arlington, VA 22207, USA; awong@marymount.edu; 7 Department of Biostatistics and Epidemiology, School of Public Health, Ahvaz Jundishapur University of Medical Sciences, Ahvaz 6135715794, Iran; m.stabib3@gmail.com; 8 Department of Nutrition, School of Medicine, Abadan University of Medical Sciences, Abadan 6313833177, Iran

**Keywords:** COVID-19, oxidative stress, total antioxidant capacity, serum superoxide dismutase, glutathione peroxidase

## Abstract

The antioxidant system can be critical in reducing exacerbated inflammation in COVID-19. This study compared the antioxidant and inflammatory responses between COVID-19 outpatients and seemingly healthy individuals. This descriptive-analytical cross-sectional study was conducted on 53 COVID-19 outpatients and 53 healthy individuals as controls. The serum concentrations of amyloid A (SAA), total antioxidant capacity (TAC), superoxide dismutase (SOD), and glutathione peroxidase (GPx) were measured and compared between COVID-19 patients and controls using the independent sample t-test before and after controlling for dietary supplement use. A generalized estimating equation (GEE) regression model, limited to COVID-19 patients, was used to evaluate the odds ratios (ORs) and 95% confidence intervals (95% CIs) of disease symptoms on days 1, 7, 14, 21, and 28 after the disease onset. Serum concentrations of SOD (*p* ≤ 0.001) and GPx (*p* = 0.001) were significantly higher in COVID-19 patients than in controls before adjustment for dietary supplement use. GPx remained significantly higher among COVID-19 patients than in controls after adjustment for all dietary supplements (*p* = 0.005)**.** Moreover, serum concentrations of GPx (*p* = 0.003), SOD (*p* = 0.022), and TAC (*p* = 0.028) remained significantly higher among COVID-19 patients than in controls after adjustment for vitamin D supplementation. This study showed higher GPx in COVID-19 outpatients than in controls after adjustment for dietary supplement use. Moreover, elevated SOD, GPx, and TAC concentrations were shown in COVID-19 outpatients compared to controls after adjusting for vitamin D supplementation. These results may provide a useful therapeutic target for treating oxidative stress in COVID-19 disease, which may help ameliorate the pandemic.

## 1. Introduction

Coronavirus disease (SARS-CoV-2) was unexpectedly identified in China in December 2019 and swiftly began to spread to other countries. The symptoms of this new type of coronavirus include fever, cough, fatigue, sore throat, and shortness of breath, which may lead to severe acute respiratory syndrome and trigger multiple organ failure and death. SARS-CoV-2 infection is often accompanied by a hyperactive immune response to the virus, which results in an excessive inflammatory reaction.

Studies have reported an association between inflammatory markers and the severity of COVID-19, although their conclusions are contradictory [1,2,3,4,5]. Several inflammatory factors, such as serum amyloid A (SAA), are used clinically for scrutinizing the clinical course of severe acute respiratory syndrome (SARS) and the rate of pneumonia in COVID-19 [6]. SAA is an acute-phase protein synthesized in response to infection, inflammation, injury, and stress [7]. A meta-analysis evaluating the association between inflammatory markers and the severity of COVID-19 showed that patients with non-severe SARS-CoV-2 infection had lower SAA concentrations than those with severe infection [3]. In addition, two studies have shown that increased SAA concentrations are associated with COVID-19 pathogenicity and may be beneficial as a potential biomarker for monitoring disease progression [8,9]. However, these results are highly controversial, as no differences in SAA concentrations were observed between different degrees of severity of SARS-CoV-2 infection [10,11]. Moreover, a prior investigation demonstrated a positive association between the oxidation–reduction potential (ORP) and SAA in critically ill patients with increased oxidative stress or a reduced antioxidant status [12].

Oxidative stress is characterized as an imbalance between reactive oxygen species (ROS) and reactive nitrogen species (RNS) production and antioxidant defenses in the human body [13], and its relationship with inflammation has been recognized by many researchers [14]. Evidence has shown that oxidative stress plays a significant pathogenic role in non-communicable diseases [15]. Damage caused by oxidative stress, such as increased lipid peroxidation and inadequate total antioxidant response, results in infectious disease and virus replication [16]. Studies on the role of oxidative stress in the pathology of SARS-CoV-2 infection have focused on the changes found in patients with COVID-19, such as its role in strengthening and sustaining cytokine storms, coagulopathy, and cell hypoxia [17]. We recently found lower glutathione reductase and higher serum concentrations of interleukin-10 (IL-10) among patients with SARS-CoV-2 infection compared to non-infected participants, indicating that impairment of redox homeostasis is responsible for ROS accumulation [18]. Consequently, the antioxidant system can be critical in mitigating the exacerbated inflammation that causes COVID-19-induced organ failure [18].

The enzymatic antioxidant system, including superoxide dismutase (SOD) and seven dependent glutathione peroxidases (GPx), is the primary endogenous enzyme defense system of all aerobic cells; these enzymes directly remove superoxide and hydrogen peroxide radicals by converting them to reactants and thereby provide protection [19]. SOD catalyzes the separation of the superoxide radical (O_2_^●−^) into hydrogen peroxide (H_2_O_2_). Although H_2_O_2_ is not a radical species, it is rapidly converted to OH radicals by the Fenton reaction, which is highly reactive [20]. One study showed that in children with acute pneumonia, enzymatic antioxidant activities associated with the antioxidant enzymes SOD and GPx and non-enzymatic enzymes associated with reduced glutathione were significantly reduced, and oxidative stress was increased [21]. However, no prior investigation has evaluated SOD and GPx levels in patients with SARS-CoV-2 infection. There is only one study that evaluated other markers of antioxidant status in this population and reported that catalase activity, serum levels of SOD, total oxidant status (TOS), and malondialdehyde (MDA) were significantly higher in cases (non-ICU and ICU patients) with SARS-CoV-2 infection compared to the control group [22]. Total antioxidant capacity (TAC) is another biomarker often used to assess oxidative stress in many pathological conditions and reflects the cumulative effect of all antioxidants existing in the blood and body fluids [23]. Serum concentrations of TAC seem to provide strong evidence of the role of free radicals (FRs) in the pathogenesis of diseases [24]. Studies on various neurodegenerative diseases, kidney disease, diabetes, and cardiomyopathy have shown a decrease in TAC compared to healthy individuals [25,26,27,28,29]. However, evidence focusing on the antioxidant and inflammatory status of outpatients with COVID-19 disease is limited. Available evidence reports lower concentrations of total antioxidant status among COVID-19 patients who require intensive care unit (ICU) support than those who do not need the ICU [30]. Furthermore, TAC activity in non-ICU COVID-19 patients did not significantly differ from that in healthy individuals [31]. Due to the insufficient evidence on the inflammatory and antioxidant status of COVID-19 outpatients and the inconsistency of available research, there is a need to assess these components. Therefore, this study aimed to investigate the underlying cause of the disease in more depth by comparing the serum concentrations of TAC, SAA, SOD, and GPx between COVID-19 outpatients and controls.

## 2. Methods and Materials

### 2.1. Participants

Our investigation was conducted on a convenience sample of people with asymptomatic or pre-symptomatic, mild, and moderate COVID-19 and age- and sex-matched non-infected participants admitted at 16-h COVID-19 health service centers with clear RT-PCR test results. Potential participants were screened based on the following inclusion/exclusion criteria. Inclusion criteria: (1) For the COVID-19 outpatient group: Individuals with a positive reverse transcription-polymerase chain reaction (RT-PCR) test for SARS-CoV-2 infection; (2) For the control group: healthy individuals with a negative RT-PCR test for SARS-CoV-2 infection. To minimize the chance of false negatives, the control group did not include individuals with COVID-19-related symptoms (such as fever and shortness of breath), individuals exposed to patients with COVID-19, or individuals in high-risk occupations (such as health care personnel, bank employees, and public transport drivers) or from pre-reading offices.

COVID-19 outpatients and controls were excluded if they were pregnant or lactating (due to the blood volume and hormonal changes and their effect on the study parameters), were <11 years of age, had uncertain RT-PCR test results, were smokers (cigarettes, hookah, etc.), consumed vitamin E supplements in the past seven days, or were unwilling to participate.

COVID-19 outpatients were categorized according to disease severity and prognosis using the Center for Disease Control and Prevention (CDC) criteria, which include the following. (1) Asymptomatic or pre-symptomatic infection: individuals who showed positive RT-PCR test results with no symptoms of COVID-19. (2) Mild illness: individuals with any of the symptoms of COVID-19 (e.g., fever, vomiting, nausea, diarrhea, muscle pain, headache, cough, sore throat, smell, taste disorders, and malaise) but no dyspnea, shortness of breath, or abnormal chest imaging. (3) Moderate illness: individuals who indicated evidence of lower respiratory disease during clinical assessment or imaging and oxygen saturation (SpO2) of ≥94% in room air at sea level [32]. None of the COVID-19 outpatients included in the study had a severe or critical illness.

The study protocol was approved by the Ethics Committee of Abadan University of Medical Sciences, Abadan, Iran, under national reference IR.ABADANUMS.REC.1399.085 on 26 August 2020. All patients were instructed on the study objectives and signed the informed consent form.

### 2.2. Study Design

This health-center-based descriptive-analytical cross-sectional study was conducted during the first wave of the COVID-19 pandemic from 6 June 2020, to 12 August 2020, in the health service centers of the Abadan University of Medical Sciences, Abadan, Iran.

Participants were assigned to one of two study groups: COVID-19 outpatients and controls. Participants in the COVID-19 outpatient group had a positive RT-PCR test for SARS-CoV-2 infection, while those in the control group had a negative RT-PCR result.

Anthropometric measurements (body mass, height, and body mass index (BMI)) and blood sampling were performed within 24 h after admission to the health center. Additionally, information on demographic characteristics (age and gender), comorbidities (e.g., cancers, diabetes mellitus, cardiovascular disease, chronic kidney disease, chronic obstructive pulmonary disease and other lung diseases, and obesity), dietary supplement use in the past seven days, symptoms of COVID-19 disease, time of onset of clinical symptoms, medical history, previous contact with infected individuals, travel history in the past two months, and current smoking status were collected in the same timeframe using a researcher-made checklist for participant self-report [33].

### 2.3. Anthropometric Measurements

Measurement of height was conducted using a commercial stadiometer (Seca©, Tokyo, Japan) with an accuracy of 0.5 cm, and weight was measured with a calibrated digital scale (Omron©, model HBF-514C scale., Technologiepark Hamburg, Germany) with a precision of 100 g. BMI was calculated by dividing weight in kilograms by the square of height in meters.

### 2.4. Blood Sampling

Within 24 h after admission to the health center, venous blood samples were collected between 8 and 10 A.M. by taking 2 ccs of blood from all participants and collected in a tube without any anticoagulant. The samples were centrifuged, and the serum was separated and stored in a freezer at −20 °C until the relevant tests.

### 2.5. Assessment of Oxidative Stress Markers

#### 2.5.1. Measurement of SAA

SAA concentrations were measured using a solid-phase sandwich ELISA method (Human ELISA Kits, ZellBio GmbH, Veltinerweg 29, D-89075, Ulm, Germany), and the absorption was read using an ELISA reader (Hiperion Co., Frankfort, Germany). The intra-assay coefficient of variation (CV) and inter-assay CV for serum SAA concentration were <10% and <12%, respectively.

#### 2.5.2. Measurement of TAC

A commercial kit (ZB-TAC-48A/ZB-TAC-96A ELISA Kit, ZellBio GmbH Co., Veltinerweg 29, D-89075, Ulm, Germany) was used to measure TAC (enzymatic and non-enzymatic compounds, such as vitamins C and E, carotene, ceruloplasmin, etc.) in serum using a microplate reader (Hiperion Co., Frankfort, Germany). In the end, a color product of the chromogenic substrate (tetramethylbenzidine) appeared. The color change was measured calorimetrically at 450 nm and expressed as millimoles/liter (mM/L). The intra-assay CV and inter-assay CV for serum TAC concentration were <3.4% and <4.2%, respectively.

#### 2.5.3. SOD Assay Method

Serum SOD was measured with the colorimetric method using a ZellBio GmbH SOD assay kit (ZB-SOD-48A/ZB-SOD-96A ELISA Kit, ZellBio GmbH Co., Veltinerweg 29, D-89075, Ulm, Germany), a microplate reader (Hiperion Co., Frankfort, Germany) at 420 nm and expressed as U/mL. The intra-assay CV and inter-assay CV for serum SOD concentration were 5.8% and 7.2%, respectively.

#### 2.5.4. GPx Assay Method

Serum GPx was measured using a ZellBio GmbH GPx assay kit based on the colorimetric assay at 412 nm (ZB-GPX-48A/ZB-GPX-96A ELISA Kit, ZellBio GmbH Co., Veltinerweg 29, D-89075, Ulm, Germany) with a microplate reader (Hiperion Co., Frankfort, Germany) and expressed as U/mL. The intra-assay CV and inter-assay CV for serum GPx concentration were ~3.5% and ~4.7%, respectively.

### 2.6. Primary and Secondary Outcome Measures and Confounders

The primary outcomes of this study were based on clinical and laboratory examinations. The secondary outcomes were related to clinical symptoms. Additionally, potential confounders were demographic variables (age, sex, education level, marital status, and smoking habits), comorbidities, BMI, and dietary supplement use.

### 2.7. Statistical Analysis

The data of the COVID-19 outpatients were matched with those of the potentially non-infected individuals in terms of sex, age, and BMI. Kolmogorov–Smirnov test was conducted to evaluate the data for normal distribution. Characteristics of COVID-19 outpatients and controls were compared using the chi-square test for discrete variables and the independent sample *t*-test for continuous variables. Furthermore, asymptomatic and mild COVID-19 outpatients were merged into a “mild and no sign” group in the data analysis.

A generalized estimating equation (GEE) regression model with a logistic link function and an exchangeable correlation structure for each individual was employed to assess the odds ratios (ORs) and 95% confidence intervals (95% CIs) of disease symptoms on days 1, 7, 14, 21, and 28 after the onset of the first symptoms. The GEE model was limited to COVID-19 patients. The model was adjusted for potential confounding variables, including age, sex, marital status, education levels, and BMI. All descriptive analyses and GEE modeling were performed using IBM SPSS Statistics (version 26). In all tests, *p* < 0.05 was considered to indicate statistical significance.

## 3. Results

### Patient Characteristics

**Figure 1:**
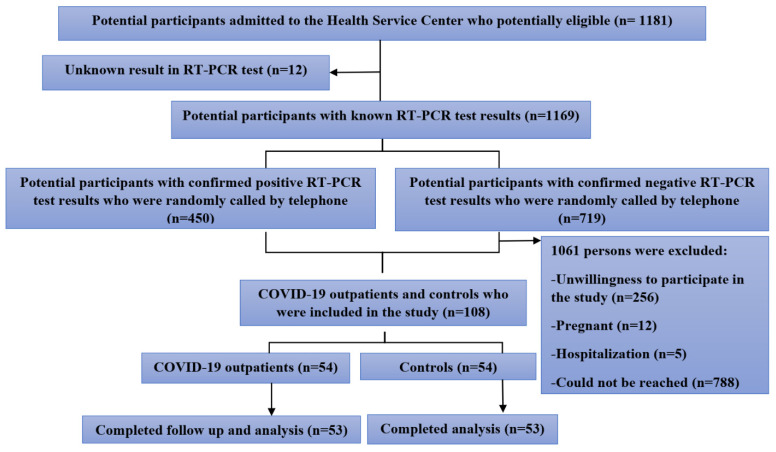
Schematic diagram of sampling.

The schematic diagram of the sampling method is illustrated in Figure 1. During the study period, 1181 potentially eligible participants were admitted to the health service center. Among them, 1169 participants were examined and confirmed to be eligible. On the basis of the inclusion criteria, a total of *n* = 54 COVID-19 outpatients and *n* = 54 controls were qualified for analysis, enrolled in the study, and started the study. However, one participant per group was excluded from the study due to a lack of response during the follow-up. Finally, *n* = 53 COVID-19 outpatients and *n* = 53 controls completed the study and finished the follow-up, providing a set of *n* = 106 consecutive serum samples. The demographic, clinical, anthropometric, and laboratory characteristics of the participants are described in Table 1. COVID-19 outpatients and age- and sex-matched controls showed similar demographic characteristics. The study included 53 COVID-19 patients (36 (67.9%) males and 17 (32.1%) females) and 53 non-infected participants (38 (71.7%) males and 15 (28.3%) females).

Moreover, a researcher-made checklist was used as a self-report to evaluate dietary supplement use in the past seven days in COVID-19 outpatients and controls as a self-report. The comparative analysis results indicated no significant differences between the two groups regarding the prevalence of vitamin C, vitamin D, zinc, and vitamin B12 supplement intakes. Specifically, 25 (47.2%) controls and 16 (30.2%) COVID-19 outpatients did not take any vitamin C supplements (*p* = 0.055); 28 (52.8%) controls and 25 (47.2%) COVID-19 outpatients did not take any vitamin D supplements (*p* = 0.349); 0 (0.00%) controls and 3 (5.7%) COVID-19 outpatients did not take any zinc supplements (*p* = 0.079); and 39 (75%) controls and 41 (77.4%) COVID-19 outpatients did not take any vitamin B12 supplements (*p* = 0.478) (data not shown in the table).

As shown in Table 1, respiratory rate (RR) (*p* = 0.001), red blood cells (RBCs) (*p* = 0.021), serum hemoglobin concentration (HGB) (*p* = 0.007), hematocrit (HCT) (*p* = 0.005), and mean platelet volume (MPV) (*p* = 0.004) were significantly higher among COVID-19 outpatients than controls. However, oxygen saturation (SpO2) (*p* = 0.032) and white blood cell count (WBC) (*p* = 0.014) were lower among COVID-19 outpatients than in controls. Moreover, positive C-reactive protein (CRP) was significantly more prevalent in COVID-19 outpatients than in controls (*p* = 0.042).

Table 2 shows that there is no significant difference between the two study groups in the prevalence of comorbidities.

**Figure 2:**
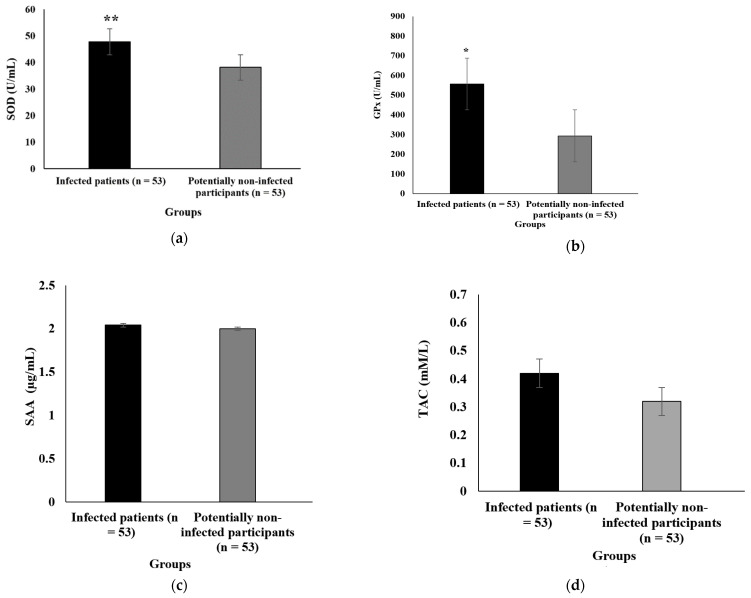
Comparison of serum concentrations of SOD (**a**), GPx (**b**), SAA (**c**), and TAC (**d**) in COVID-19 outpatients and controls before controlling for dietary supplement use. An independent sample *t*-test was applied to analyze data. SOD and GPx were significantly higher among COVID-19 outpatients than in controls. GPx, glutathione peroxidase; SAA, serum amyloid A; SOD, superoxide dismutase; TAC, total antioxidant capacity. * *p* = 0.001, ** *p* ≤ 0.001.

Figure 2 illustrates the oxidative stress levels of all participants before adjustment for dietary supplement use. There was a statistical difference between COVID-19 outpatients and controls regarding SOD and GPx concentrations. SOD (*p* ≤ 0.001) and GPx (*p* = 0.001) were markedly higher in COVID-19 outpatients than in controls before controlling for dietary supplement use. However, no statistical difference in SAA and TAC concentrations was found between groups.

**Figure 3:**
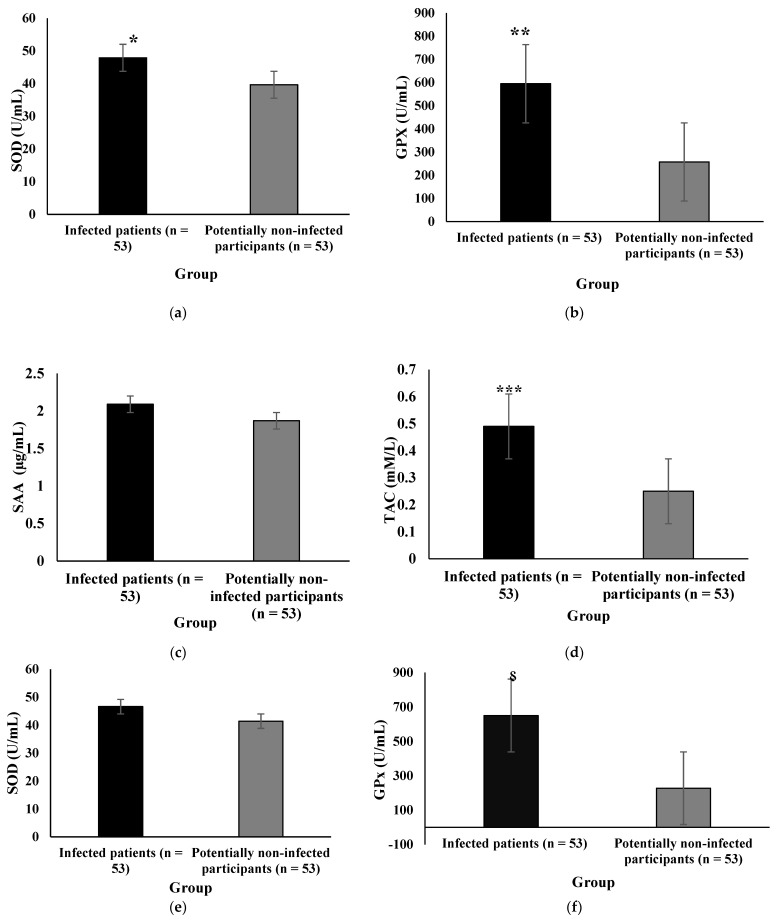

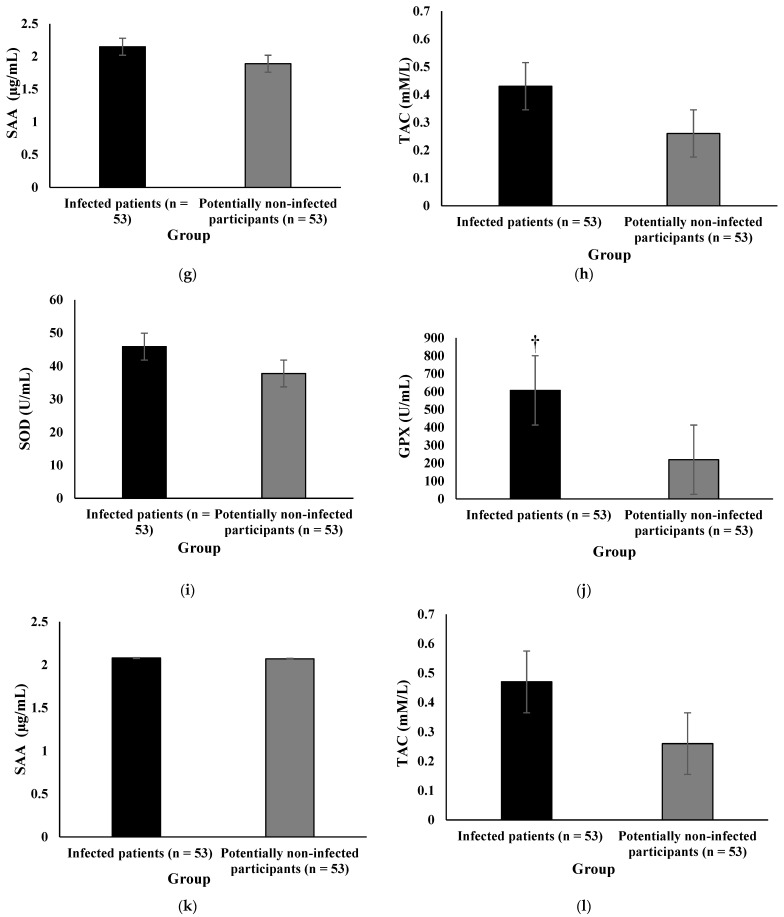
Comparison of SOD, GPx, SAA, and TAC serum concentrations in COVID-19 outpatients and controls after adjusting for the use of vitamin D supplements (**a**–**d**), all dietary supplements (**e**–**h**), and vitamin C supplements (**i**–**l**). An independent sample *t*-test was applied to analyze data. SOD (**a**), GPx (**b**), and TAC (**d**) concentrations were significantly higher among COVID-19 outpatients than in controls after adjusting for the use of vitamin D supplements. GPx was significantly higher among COVID-19 outpatients than in controls after adjusting for vitamin C (**j**) and all dietary supplements (**f**). GPx, glutathione peroxidase; SAA, serum amyloid A; SOD, superoxide dismutase; TAC, total antioxidant capacity. * *p* = 0.003, ** *p* = 0.022, *** *p* = 0.028, ^⸹^ *p* = 0.005, and † *p* = 0.003.

Figure 3 illustrates the oxidative stress levels of all participants after adjusting for the use of antioxidant and anti-inflammatory supplements. Serum concentrations of SOD (Figure 3a, *p* = 0.022), GPx (Figure 3b, *p* = 0.003), and TAC (Figure 3d, *p* = 0.028) remained significantly higher among COVID-19 outpatients than controls after adjusting for the use of vitamin D supplements. However, there was no statistical difference in SAA between groups after adjusting for vitamin D supplementation (Figure 3c, *p* = 0.102). Additionally, GPx (Figure 3j, *p* = 0.003) remained significantly higher among COVID-19 outpatients than controls after adjusting for vitamin C supplementation. However, there was no statistical difference in SOD (Figure 3i, *p* = 0.128), SAA (Figure 3k, *p* = 0.955), and TAC (Figure 3l, *p* = 0.084) between the two groups after adjusting for the use of vitamin C supplements. In addition, GPx remained significantly higher among COVID-19 outpatients than controls even after adjusting for the use of all dietary supplements (Figure 3f, *p* = 0.005). However, there was no statistical difference between the two groups regarding serum concentrations of SOD (Figure 3e), SAA (Figure 3g), and TAC (Figure 3h) after adjusting for the use of all dietary supplements.

Comparisons of serum concentrations of SOD, GPx, SAA, and TAC between COVID-19 patients with different disease severities are reported in Table 3. Our results showed that for all study parameters, there were no significant differences between COVID-19 patients in the mild and asymptomatic group compared to those with moderate-grade severity.

Table 4 shows the results of the GEE model for the longitudinal relationship between oxidative stress and antioxidant status from 1 to 28 days after disease onset and its clinical symptoms adjusted for age, sex, marital status, education levels, and BMI among individuals with COVID-19, as confirmed by a positive PCR result for SARS-CoV-2. The results revealed that the odds ratios of pulmonary symptoms of COVID-19 in patients with less than high school education and those with college education were 0.06 and 0.07 times than that of illiterate patients (OR = 0.06; 95% CI, 0.005–0.66; *p* = 0.022 and OR = 0.07; 95% CI, 0.006–0.98; *p* = 0.048, respectively). Furthermore, married patients were more likely to experience gastrointestinal symptoms than single patients, with an OR of 4.48 (95% CI, 1.28–15.67; *p* = 0.022). Additionally, the odds ratio of neurologic symptoms of COVID-19 was 0.4 times lower in males than in females (OR = 0.40; 95% CI, 0.17-0.94; *p* = 0.016). Additionally, the odds ratio of neurologic symptoms of COVID-19 in patients with less than high school education was 0.2 times lower than that of illiterate patients (OR = 0.2; 95% CI, 0.28–0.97; *p* = 0.046). For every unit increase in BMI, the risk of developing neurological symptoms increased one-fold (OR = 1.1; 95% CI, 1.01–1.21; *p* = 0.025). All symptoms showed a decreasing trend over time. However, age and oxidative stress markers were not significantly associated with clinical symptoms (*p* ≥ 0.05).

## 4. Discussion

To our knowledge, this is the first investigation to compare serum concentrations of SOD, GPx, SAA, and TAC between COVID-19 outpatients and controls. We observed that COVID-19 outpatients had higher SOD and GPx than controls. These results remained significant for GPx after adjusting for all dietary supplements. Moreover, serum concentrations of SOD and TAC remained significantly higher among COVID-19 outpatients than in controls after adjusting for vitamin D supplements.

Several studies have suggested an association between oxidative stress and COVID-19 pathogenesis [22,30,31]. In a hospital-based cross-sectional study, Mehri et al. reported that COVID-19 patients had higher serum concentrations of SOD and GPx than healthy participants [22]. On the contrary, a study on COVID-19-infected inpatients classified into ICU and non-ICU groups revealed that an increase in oxidative stress and a decrease in antioxidant activity among patients were associated with worse disease [31]. They reported no significant difference in total oxidant status (TOS) serum level between ICU and non-ICU COVID-19 patients. However, small sample sizes reduced the power of both studies [22,31]. SOD and GPx are both zinc-dependent antioxidant proteins. In COVID-19 disease, intracellular zinc depletion causes the dysfunction of zinc-dependent antioxidant proteins such as SOD and GPx, which consequentially induces ROS overproduction, resulting in further exacerbation of oxidative stress [34]. Additionally, since SOD and GPx are considered the most important mechanisms of ROS and RNS detoxification [35], the high serum levels of antioxidant enzymes shown in this study may be a remedial mechanism to neutralize the oxidative stress observed in SARS-CoV-2 infection [22]. These results highlight SOD and GPx as critical components over other antioxidant enzymes for oxidative stress regulation in COVID-19 disease.

In the present study, positive CRP was more prevalent among COVID-19 outpatients than in controls. A similar result was reported in a study by Mehri et al., where mean CRP concentrations were significantly higher among COVID-19 patients admitted to the ICU compared to non-ICU patients and healthy participants [22]. Indeed, a rise in CRP concentration has been previously associated with an increase in COVID-19 disease severity [36]. This association was further supported by a retrospective single-center study in Wuhan, China, in which patients with severe COVID-19 disease showed significantly higher CRP levels than their non-severe counterparts [37].

We also found that serum concentrations of SAA were not significantly different between COVID-19 outpatients and controls. CRP and SAA, which are released by hepatocytes after cytokine stimulation, are frequently used to predict, diagnose, and evaluate many inflammatory diseases [38,39,40]. SAA is a non-specific acute-phase protein mainly produced by the cytokines interleukin-1β (IL-1β), interleukin-6 (IL-6), and tumor necrosis factor-α (TNF-α) in the liver [41]. Specific attention has been paid to SAA, which is significantly elevated in patients with bacterial infections compared to those with viral infections. This increase is positively associated with CRP concentration, and some have considered SAA to be comparable to CRP in clinical practice, although SAA might be a more sensitive laboratory indicator than CRP in infections with low inflammatory activity [42,43]. While the use of SAA as a biomarker for COVID-19 requires further research, CRP and SAA are commonly used in conjunction to monitor inflammatory diseases [6,38]. Although the patients in our study were asymptomatic or had mild or moderate illness, prior research indicates that SAA increases in severe COVID-19 infection [44]. Nevertheless, further research studies with large sample sizes are necessary to establish the practical significance and application of SAA as a COVID-19 biomarker [45].

Our outcomes indicated that the serum concentration of TAC was significantly higher among COVID-19 outpatients than controls after adjusting for vitamin D supplementation. It has been observed that serum concentrations of TAC increase in ICU hospitalized COVID-19 patients with and without endotracheal intubation compared to healthy people without COVID-19 disease [31]. In general, viruses cause an imbalance in the cellular redox environment, which, depending on the virus and the cell, can result in different responses, e.g., cell signaling and antioxidant defenses [46]. When a virus enters the human body under oxidative stress conditions, the levels of antioxidant biomarkers increase to protect against oxidative compounds [31]. On the other hand, existing evidence shows that the adequate intake of specific foods and nutrients can effectively improve the inflammatory state and oxidative stress [47]. These concepts emphasize the importance of the relationship between nutrition, inflammation, oxidative stress, and the body’s response to viral infections. Consequently, the poor nutritional status of zinc is associated with an increased risk of COVID-19 disease, as shown in our recent study [48].

COVID-19 outpatients in our study also had significantly higher RR, RBC count, HGB concentration, HCT, and MPV than the controls. In addition, COVID-19 outpatients had lower SpO2 and WBC counts compared to the controls. One study showed that blood laboratory parameters could be used to predict COVID-19 disease. In line with our findings, previous reports have shown that patients with positive COVID-19 RT-PCR tests had a lower WBC count than individuals with negative results [49,50]. Thus, these results indicate the accurate screening of SARS-CoV-2-infected patients in the present study.

Our investigation has various strengths, including the adjustment of the results based on dietary supplement use. Since dietary ingredients with especially high anti-inflammatory and antioxidant capacity can interact with transcription factors, such as nuclear factor kappa B (NF-kB), activating protein-1 (AP-1), and nuclear factor erythroid 2-related factor 2 (Nrf-2), they inhibit virus particles from entering the cell by interacting with the cellular receptor for viral entry, i.e., angiotensin-converting enzyme 2 (ACE2) [51,52]. This may reduce inflammation and oxidative stress, thereby strengthening the immune system during the COVID-19 crisis [53,54]. Another strength of our work is the longitudinal study design, which allowed us to estimate the relationships between the progression of observed COVID-19 symptoms with demographic, BMI, and laboratory parameters among patients who were PCR positive for SARS-CoV-2.

Despite the above-mentioned strengths of our study, some limitations should be noted. First, the sample sizes were relatively small; therefore, our results cannot be generalized and require confirmation via large-scale investigations. Second, a detailed analysis of the antioxidant effects of the parameters mentioned in the study could not be performed or related to the course of the disease unless more inflammatory factors and cytokines were measured. Third, dietary supplement use is considerably associated with participants’ socioeconomic status and lifestyle factors such as age, sex, marital status, educational levels, cigarette smoking, and BMI. Chronic health conditions such as cancer, cardiovascular disease, hypertension, or diabetes may stimulate dietary supplement use. However, to minimize the chance of outstanding confounding factors, we carefully adjusted for all of these factors in the GEE model. In addition, we compared the demographic, clinical, and anthropometric characteristics and the presence of comorbid conditions between COVID-19 outpatients and non-infected participants at baseline, and the characteristics and conditions remained similar. Fourth, dietary supplement use was assessed in the previous seven days, which may not reflect regular supplement use. Moreover, the prevalence of supplement use was also based on self-report and subject to recall bias. However, there was no significant difference between COVID-19 outpatients and non-infected participants in the prevalence of dietary supplement use, reducing the analysis error due to recall bias. Lastly, we did not evaluate certain pro-inflammatory cytokines (e.g., IL-1, IL-6, IL-18, IFN-γ, and TNF-α) and biomarkers of oxidative damage to biomolecules (e.g., MDA and protein carbonyls), which would have strengthened our results. Future investigations should focus on the assessment of these markers in outpatients with SARS-CoV-2 infection.

## 5. Conclusions

In conclusion, we observed a higher concentration of GPx in outpatients with SARS-CoV-2 infection compared to controls after adjusting for dietary supplements. Moreover, it is worth noting that COVID-19 outpatients also had greater serum concentrations of SOD and TAC than controls after adjusting for vitamin D supplementation. Our results will be able to assist in developing potential physio-pathological pathways involved in SARS-CoV-2 infection and may help physicians and dietitians find complementary therapies and antiviral medications to improve antioxidant and inflammatory status in COVID-19 disease.

## Figures and Tables

**Table 1:** Demographic, clinical, anthropometric, and laboratory characteristics of patients infected with COVID-19 and matched controls at baseline ^†^.

Characteristics		COVID-19 Outpatients (*n* = 53)	Controls (*n* = 53)	*p*-Value
Age (year)		41 ± 12.9	40 ± 13.4	0.609
	<50	40 (18.4%)	42 (79.2%)	
	50–64	9 (17.6%)	9 (17.0%)	0.995
	≥65	2 (3.9%)	2 (3.8%)	
Sex				
	Female	17 (32.1%)	15 (28.3%)	
	Male	36 (67.9%)	38 (71.7%)	0.416
Married status				
	Single	12 (22.6%)	12 (22.6%)	0.592
	Married	41 (77.4%)	41 (77.4%)	
Education levels				
	Illiterate	2 (3.8%)	1 (1.9%)	0.754
	Less than high school	15 (28.8%)	19 (36.8%)	
	High school	13 (25%)	10 (19.2%)	
	College education	22 (42.3%)	22 (42.3%)	
Cigarette smoking				
	No	40 (75.5%)	45 (84.9%)	0.165
	Yes	13 (24.5%)	8 (15.1%)	
RR (number/min)		14.1 ± 1.62	13 ± 1.9	0.001
PR (number/min)		90.71 ± 18	87.23 ± 13.3	0.271
SpO2 (%)		96.9 ± 1.35	97.4 ± 1.23	0.032
Duration of infection (day)		6.5 ± 2	-	
BMI (kg/m^2^)		27.1 ± 4.7	27.5 ± 4.3	0.663
CRP				
	Negative	37 (69.8%)	48 (90.6%)	0.007
	Positive	16 (30.2%)	5 (9.4%)	
WBC, 10 × 3/µL		6 ± 1.3	6.8 ± 2	0.014
RBC, 10 × 3/µL		4.8 ± 0.6	4.6 ± 0.5	0.021
HGB, g/dL		14 ± 1.4	13.2 ± 1.6	0.007
HCT, %		41 ± 4.1	38.7 ± 4.1	0.005
MCV, fL		85.1 ± 6.7	85 ± 6.8	0.939
MCH, pg		29.3 ± 2.5	29 ± 2.8	0.593
MCHC, g/dL		34.4 ± 0.8	34.1 ± 1.3	0.114
RDW, %		13.1 ± 1	13.5 ± 1.1	0.109
MPV, %		9.8 ± 1.1	9.2 ± 0.9	0.004
PDW, %		16.9 ± 0.5	17.1 ± 1.6	0.398
Platelets		250.1 ± 72.2	236.6 ± 64.8	0.312
NEU, %		52.9 ± 8.5	52.2 ± 9.4	0.657
LYM, %		42.4 ± 8.8	43.3 ± 9.2	0.612
MNC, %		3.7 ± 1.2	3.4 ± 1.3	0.285
EOS, %		1.4 ± 0.6	1.5 ± 0.6	0.574

^†^ An independent sample *t*-test was conducted to analyze continuous variables, and the results are expressed as mean ± standard deviation. Categorical variables were analyzed by chi-square test, and the results are presented as number (%). BMI, body mass index; CRP, C-reactive protein; EOS, eosinophil; HCT, hematocrit; HGB, hemoglobin; LYM, lymphocyte; MCH, mean corpuscular hemoglobin; MCHC, mean corpuscular hemoglobin concentration; MCV, mean corpuscular volume; MNC, monocyte; MPV, mean platelet volume; NEU, neutrophil; RBC, red blood cell count; PDW, platelet distribution width; PLT, platelet count; RDW: RBC distribution width; RR, respiratory rate; PR, pulse rate; SpO2, oxygen saturation; WBC, white blood cell count.

**Table 2:** Comorbidities of patients infected with COVID-19 and matched controls at baseline ^†^.

Comorbidities		COVID-19 Outpatients (*n* = 53)	Controls (*n* = 53)	*p*-Value
Hypertension, *n* (%)				
	Yes	10 (18.9)	5 (9.4)	
	No	43 (81.1)	48 (90.6)	0.164
Type 2 diabetes mellitus, *n* (%)				
	Yes	6 (11.3)	4 (7.5)	
	No	47 (88.7)	49 (92.5)	0.506
Obesity, *n* (%)				
	Yes	13 (24.5)	21 (39.6)	
	No	40 (75.5)	32 (60.4)	0.096
Malnutrition, *n* (%)				
	Yes	1 (1.9)	0 (0.00)	
	No	52 (98.1)	53 (100)	0.096
Asthma and allergy, *n* (%)				
	Yes	6 (11.3)	5 (9.4)	
	No	47 (88.7)	48 (90.6)	0.500
Cancer, *n* (%)				
	Yes	2 (3.8)	0 (0.00)	
	No	51 (96.2)	53 (100)	0.462
Chronic pulmonary disease, *n* (%)				
	Yes	2 (3.8)	0 (0.00)	
	No	51 (96.2)	53 (100)	0.153
Chronic neurological disease, *n* (%)				
	Yes	2 (3.8)	1 (1.9)	
	No	50 (96.2)	52 (98.1)	0.547
Chronic hematological disease, *n* (%)				
	Yes	2 (3.8)	0 (0)	
	No	51 (96.2)	52 (100)	0.157
Liver disease, *n* (%)				
	Yes	5 (9.4)	3 (5.7)	
	No	48 (90.6)	50 (94.3)	0.462
Renal disease, *n* (%)				
	Yes	3 (5.7)	4 (7.5)	
	No	50 (94.3)	49 (92.5)	0.696
Chronic heart disease, *n* (%)				
	Yes	4 (7.5)	2 (3.8)	
	No	49 (92.5)	50 (96.2)	0.414
HIV, *n (%)*				
	Yes	2 (3.8)	0 (0.00)	
	No	51 (96.2)	53 (100)	0.153
Rheumatoid arthritis (RA) *n* (%)				
	Yes	1 (1.9)	1 (1.9)	
	No	52 (98.1)	52 (98.1)	1.00
Other diseases *				
	Yes	8 (15.1)	16 (30.2)	
	No	45 (84.9)	37 (69.8)	0.063

^†^ An independent sample *t*-test was conducted to analyze continuous variables, and the results are expressed as mean ± standard deviation. Categorical variables were analyzed by chi-square test, and the results are presented as number (%). * Other diseases include autoimmune disease, hemoglobinopathies, migraine, digestive system problems, hypothyroidism, hyperthyroidism, hyperlipidemia, endometrioses, and neck and back disk issues.

**Table 3:** Differences in the study parameters between patients who had different disease severities *.

Parameters	Mild and Asymptomatic	Moderate	*p*-Value
SOD (U/mL)	47.12 ± 16	49.74 ± 11.84	0.523
GPX (U/mL)	533.85 ± 436.38	616.18 ± 395.59	0.541
SAA (µg/mL)	1.99 ± 0.39	2.21 ± 0.53	0.227
TAC (mM/L)	0.46 ± 0.34	0.32 ± 0.37	0.232

* The data were analyzed by an independent sample *t*-test. GPx: glutathione peroxidase; SAA: serum amyloid A; SOD: superoxide dismutase; TAC: total antioxidant capacity.

**Table 4:** Estimates of observed symptom progression of COVID-19 and the association with demographic, BMI, and laboratory parameters among COVID-19 outpatients (*n* = 53) ^†^.

		Symptom Categories			
Parameters		General	Pulmonary	Gastrointestinal	Neurologic
Age (year)		0.99 (0.96–1.03)	0.96 (0.90–1.02)	0.96 (0.91–1.01)	0.97 (0.93–1.00)
Sex					
	Female	1 (ref)	1 (ref)	1 (ref)	1 (ref)
	Male	0.73 (0.34–1.56)	1.51 (0.48–4.73)	0.81 (0.42–1.56)	0.40 (0.17–0.94) *
Married status					
	Single	1 (ref)	1 (ref)	1 (ref)	1 (ref)
	Married	2.13 (0.49–9.26)	0.82 (0.22–3.00)	4.48 (1.28–15.67) *	1.53 (0.39–5.95)
Education levels					
	Illiterate	1 (ref)	1 (ref)	1 (ref)	1 (ref)
	Less than high school	0.81 (0.16–4.12)	0.06 (0.005–0.66) *	0.26 (0.04–1.50)	0.10 (0.02–0.62) *
	High school	0.66 (0.12–3.73)	0.22 (0.02–2.78)	0.29 (0.03–2.90)	0.22 (0.04–1.08)
	College education	1.01 (0.22–4.73)	0.07 (0.006–0.98) *	0.66 (0.10–4.42)	0.29 (0.07–1.26)
SOD		0.97 (0.94–1.00)	1.00 (0.95–1.06)	0.99 (0.96–1.02)	1.00 (0.97–1.03)
GPx		1.00 (0.999–1.00)	1.00 (1.00–1.001)	1.00 (0.999–1.00)	1.00 (0.999–1.001)
SAA		0.27 (0.07–1.06)	2.31 (0.68–7.90)	0.44 (0.13–1.47)	0.37(0.11–1.17)
TAC		0.53 (0.08–3.68)	1.41 (0.25–7.99)	2.41 (0.48–12.22)	0.55 (0.09–3.40)
BMI (kg/m^2^)		1.07 (0.99–1.15)	0.96 (0.87–1.05)	1.07 (0.98–1.18)	1.11 (1.01–1.21) *

^†^ Odds of common clinical signs and symptoms of COVID-19 from days 1 to 28 of disease onset (95% CI *). A general estimation equation (GEE) was applied to analyze data. The working correlation matrix structure was exchangeable. *: *p* < 0.05.

## Data Availability

The data presented in this study are available on request from the corresponding author.
